# Supplementary material for: Three-dimensional scanless holographic optogenetics with temporal focusing (3D-SHOT)
Source: Nat Commun. 2017 Oct 31;8:1228. doi: 10.1038/s41467-017-01031-3 (PMC5663714; doi:10.1038/s41467-017-01031-3)
Supplement: Supplementary file 1 — Supplementary Information [file 41467_2017_1031_MOESM1_ESM.pdf]

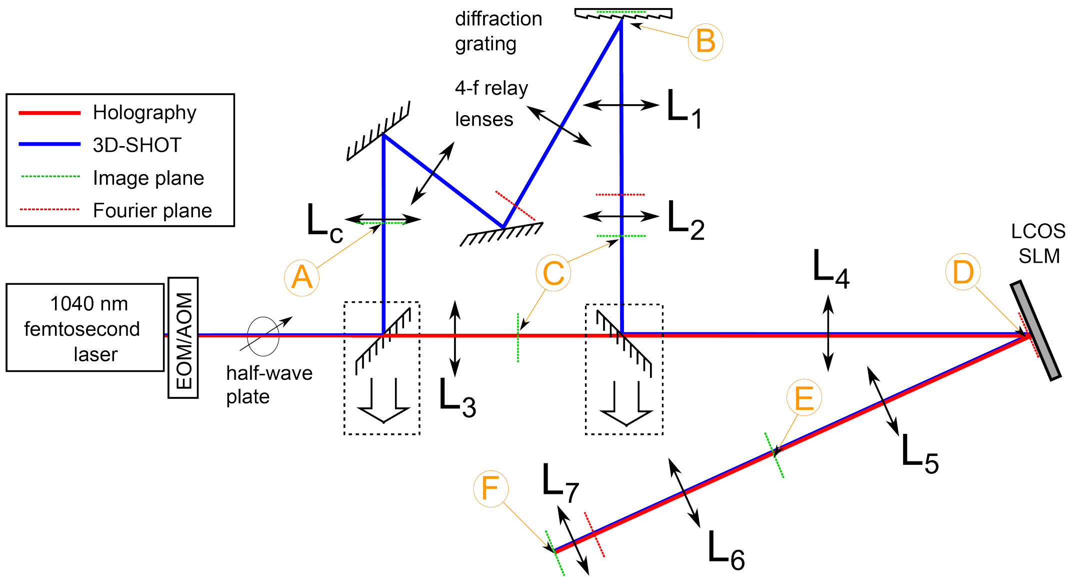

Supplementary Figure 1: **Experimental setup**. For conventional holography (red path, Supplementary Note 2.), a femtosecond laser beam is expanded with lenses, L_3, and L_4 onto a phase-only SLM. We compute a phase mask in the Fourier domain that best replicates a custom 3D intensity distribution after propagation to the real space through the telescope lens, L_5. The resulting volume hologram is then demagnified with the microscope tube lens, L_6, and objective L_7. For 3D-SHOT (blue path, Supplementary Note 3.), a diffraction grating decomposes laser pulses in the spectral domain, and phase patterning, here with an additional lens, L_C, enables digital holography in the Fourier domain. We target the desired locations in 3D with point-cloud holography (Supplementary Note 6.). All-optical convolution replicates the same temporally-focused pattern on targeted neurons for efficient photo-excitation with high spatial specificity.

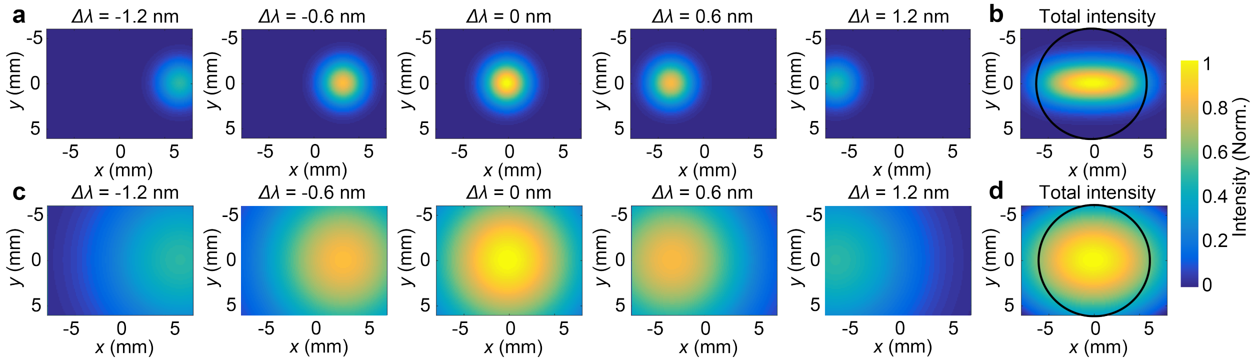

Supplementary Figure 2: **Intensity spectral distribution at SLM plane.** (a,c) $|A_{\mathrm{D}}(x, y, \Delta k = (2\pi\Delta\lambda/\lambda_0^2))|^2$ is displayed for various values of $\Delta\lambda$ within the wavelength spectrum range of a time-bandwidth limited femtosecond pulse and in a window corresponding to the SLM area. (a) Without applying a spherical phase mask to the field before the diffraction grating, the SLM is at the image plane of a diffraction limited point, swept along the grating direction. (b) The corresponding intensity pattern on the SLM, a focused line, is reflected by a much smaller number of pixels (less than 7% of the SLM pixels are above Half-Max intensity threshold) and taking advantage of the SLM wavefront beam shaping capabilities for holography remains impossible. (c) With lens L_{C} in place, an appropriate amount of curvature to the incoming wavefront allows for a more uniform spreading of the wavefront onto the SLM for all values of $\Delta\lambda$ within the pulse bandwidth. (d) Corresponding intensity pattern on SLM. The circle represents the back-aperture of the microscope objective and the effective area of the SLM.

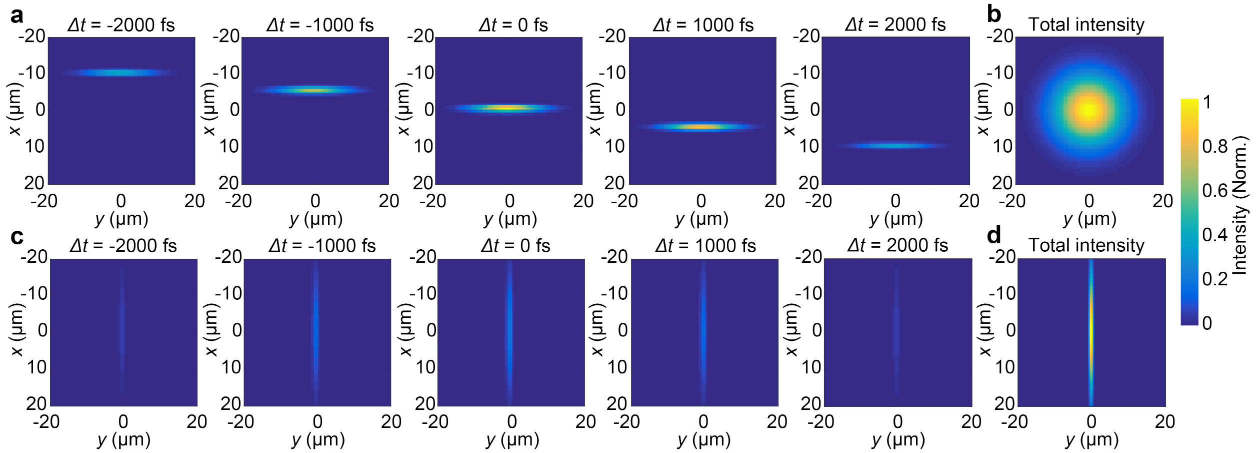

Supplementary Figure 3: **Two-photon absorption in primary and in secondary focus.** (a) Primary focus two-photon absorption $I_{\mathrm{F}}^2(x, y, \Delta t, z = 0)$ is simulated for various values of Δt, and shows temporal focusing propagating long the $\boldsymbol{x}$ axis. (b) Time-averaged two-photon absorption at primary focus: $I_{\mathrm{F}}^2(x, y, z = 0)$. (c) Two-photon absorption in the secondary focus $I_{\mathrm{F}}^2(x, y, \Delta t, z = 25)$ μm for various values of Δt, shows a static geometric focus line broadened in the time domain. (d) Time-averaged two-photon absorption at secondary focus: $I_{\mathrm{F}}^2(x, y, z = 25\ \mu\mathrm{m})$.

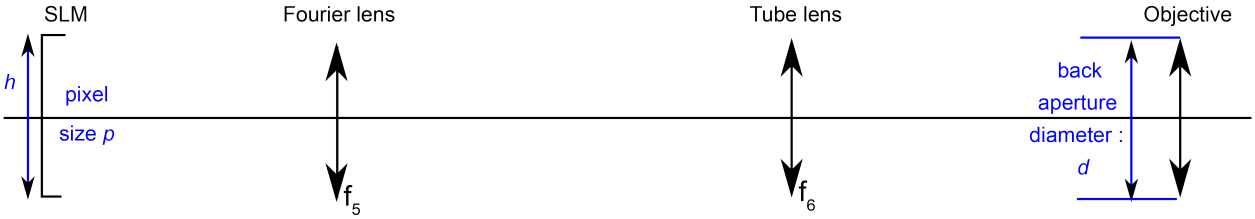

Supplementary Figure 4: **Simplified diagram of the optical system (hologram synthesis section)**. We define the effective area of the SLM, h, and the pixel size, p. The Fourier lens, tube lens, and objective, and their respective focal lengths, and d, the back-aperture diameter of the objective.

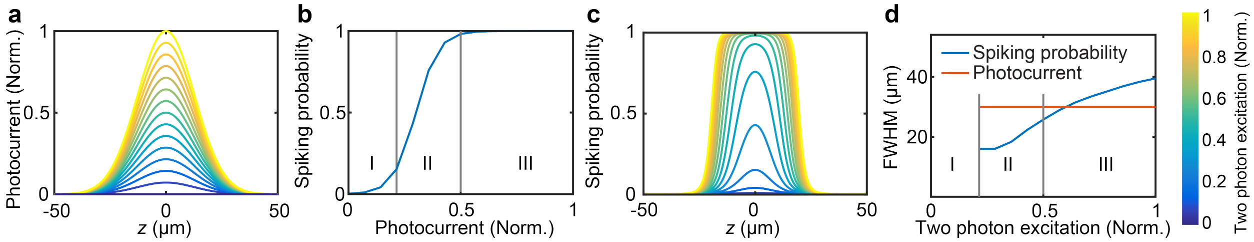

Supplementary Figure 5: **Biological features determine the optimal power level for neuron photoactivation** (a) We consider a Gaussian model, with Full-Width-Half-Max : $\mathrm{FWHM} = 30$ μm to represent photocurrents induced in a neuron at various power levels. (b) We use a sigmoidal response profile to model the statistical distribution of spike probability as a function of induced photocurrent. We identify three characteristic zones, (I) below, (II) near, and (III) above threshold. (c) The resulting spatial distribution of spike probability shows two regimes with a compromise between maximal spiking probability at $z = 0$ and spatial resolution. (d) Simulation results show how the FWHM of the spiking probability distribution increases as unnecessary additional optical power is sent onto the neuron.

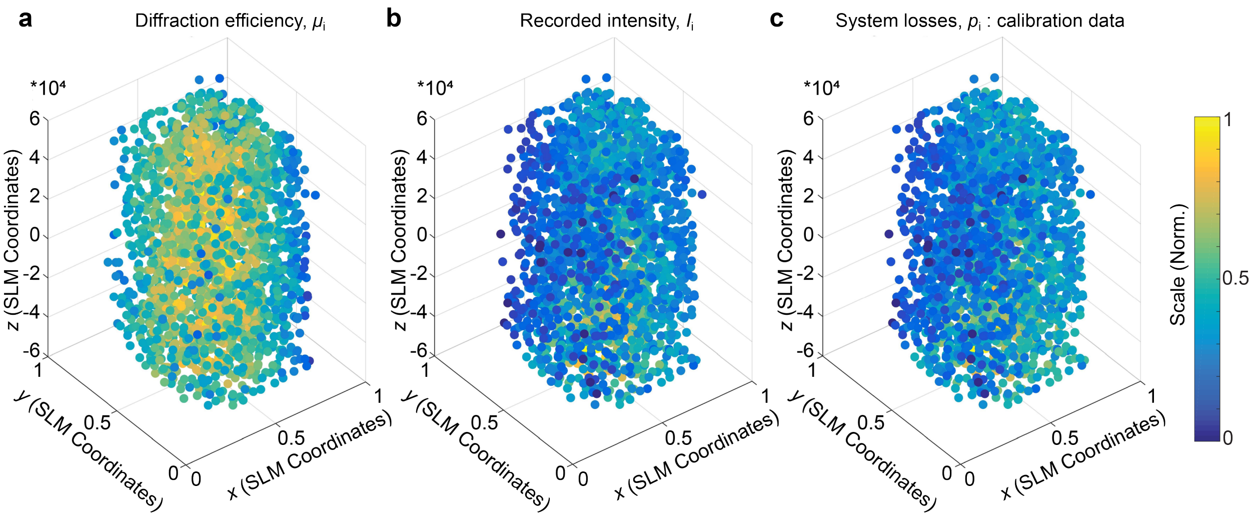

Supplementary Figure 6: **Power calibration (15000 points, 3000 shown).** (a) Computed diffraction efficiency, μ_i, from digital simulation. (b) Normalized recorded intensity, I_i, from single target point holograms acquired during the calibration experiment. (c) System specific optical loss, p_i, to be used as interpolation data points for power correction in subsequent experiments.

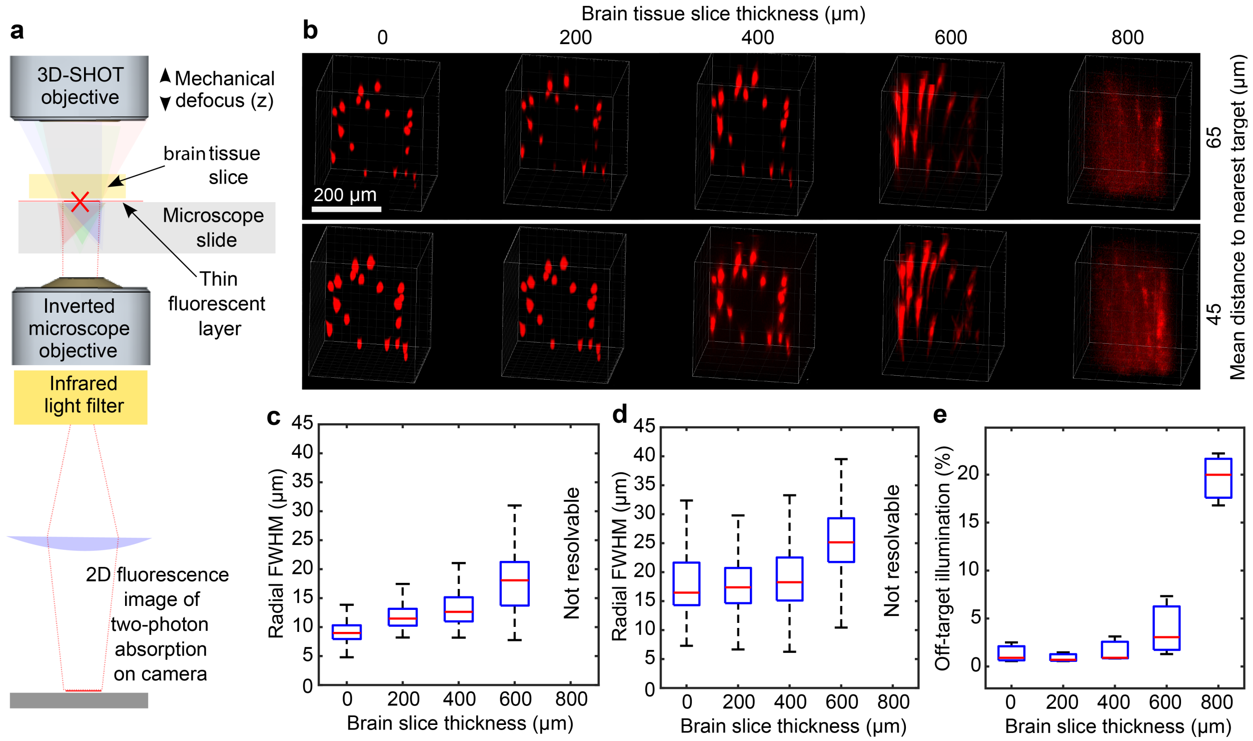

Supplementary Figure 7: Spatial resolution through mouse brain tissue. (a) Experimental setup with an inverted fluorescence microscope allowing 3D recording of two-photon absorption through custom thicknesses of brain tissue slice. (b) Volumetric recordings of two photon absorption through increasing thickness of mouse brain tissue for the same hologram. (c) Radial, and (d), radial FWHM as a function of scattering depth shows accumulated effects of optical propagation through brain tissue. (e) Amount of light received in non-targeted locations as a function of scattering depth shows a clear degradation of hologram quality below 500 µm.

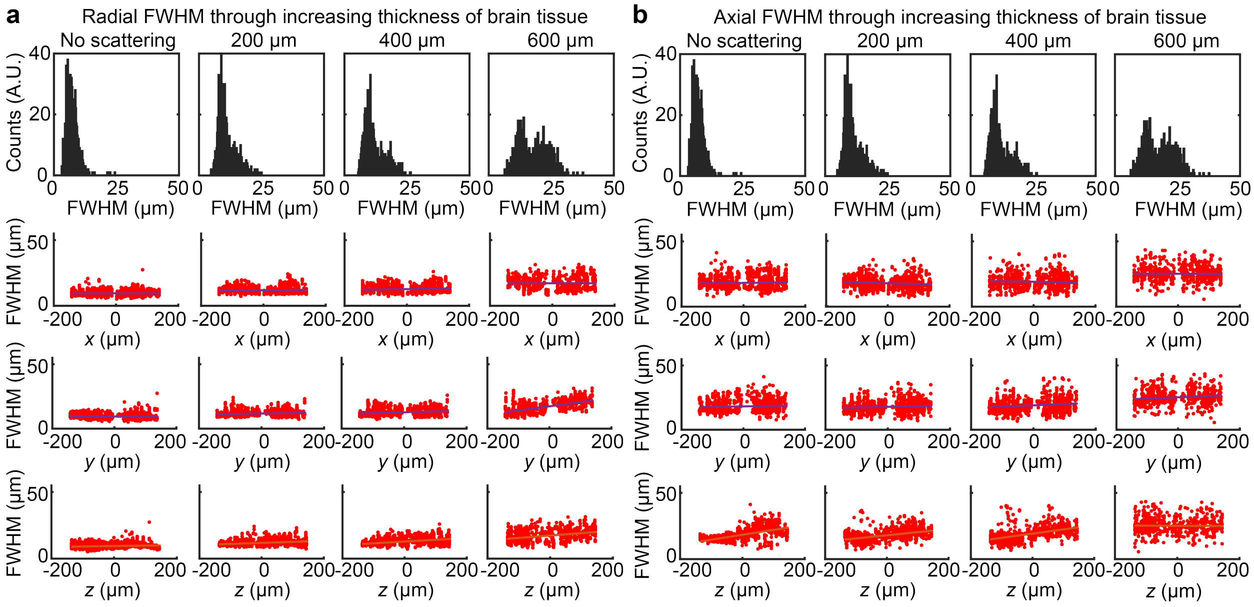

Supplementary Figure 8: **Spatial resolution through mouse brain tissue.** (a) radial, and (b) axial FWHM measured for various holograms with randomized spatial distributions of 20 to 75 targets as a function of scattering depth through mouse brain tissue, from left (0 µm) to right (600 µm)). Histogram data distributions are shown in black. In each case, we also show scatter plots (in red) to display the FWHM as a function of the x,y,z location of each target within the hologram .

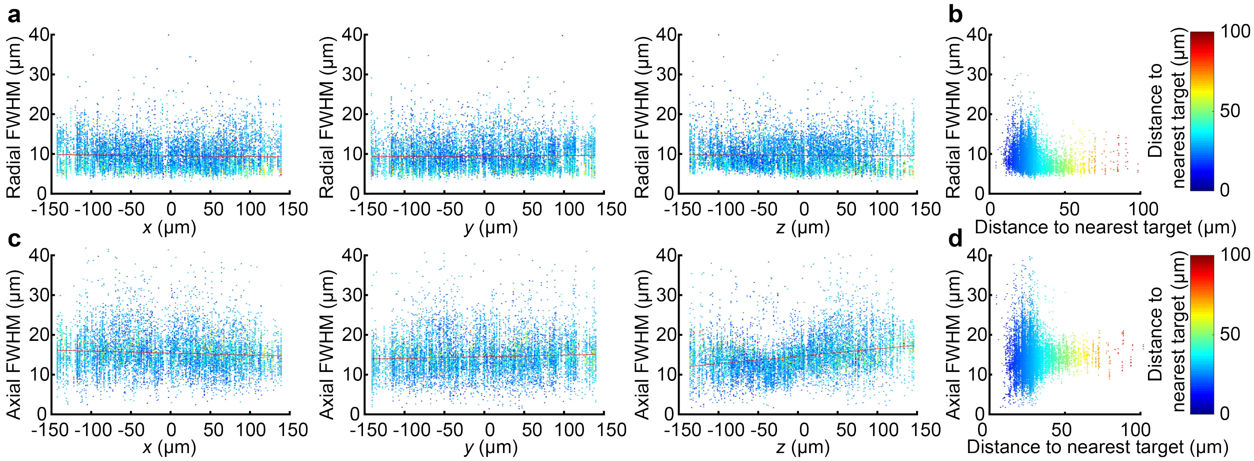

Supplementary Figure 9: **Spatial resolution and 3D target position.** (a) Radial FWHM of multi-target holograms as a function of target location. Color coding indicates distance to nearest target. (b) - Same as in (a) but by displaying radial FWHM as a function of target distance to the nearest neighbor. (c), (d) : same as in (a), resp. (b) but with axial FWHM.

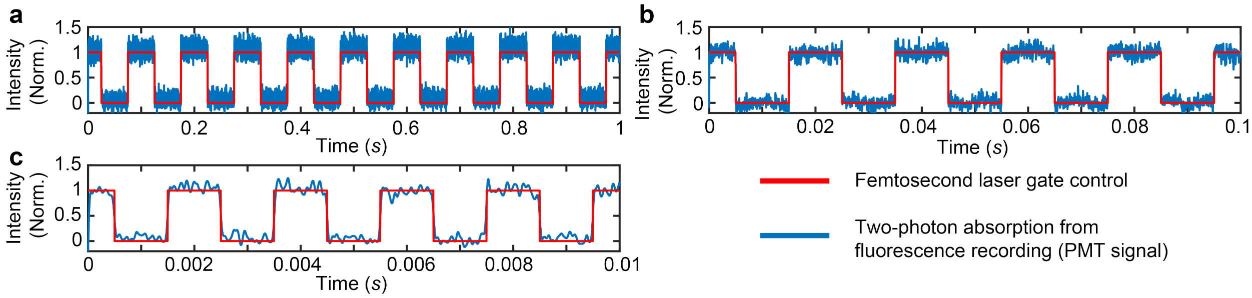

Supplementary Figure 10: **Temporal response of the optical system.** The laser gate was triggered at various frequencies ((a) 10 Hz,(b) 100 Hz,and (c) 1000Hz). A hologram was placed on the SLM to illuminate a spot in a fluorescent slide. A photomultiplier tube (PMT) synchronously recorded the fluorescence induced by two-photon absorption. Results show no detectable delay for operations at speeds below 1KHz.

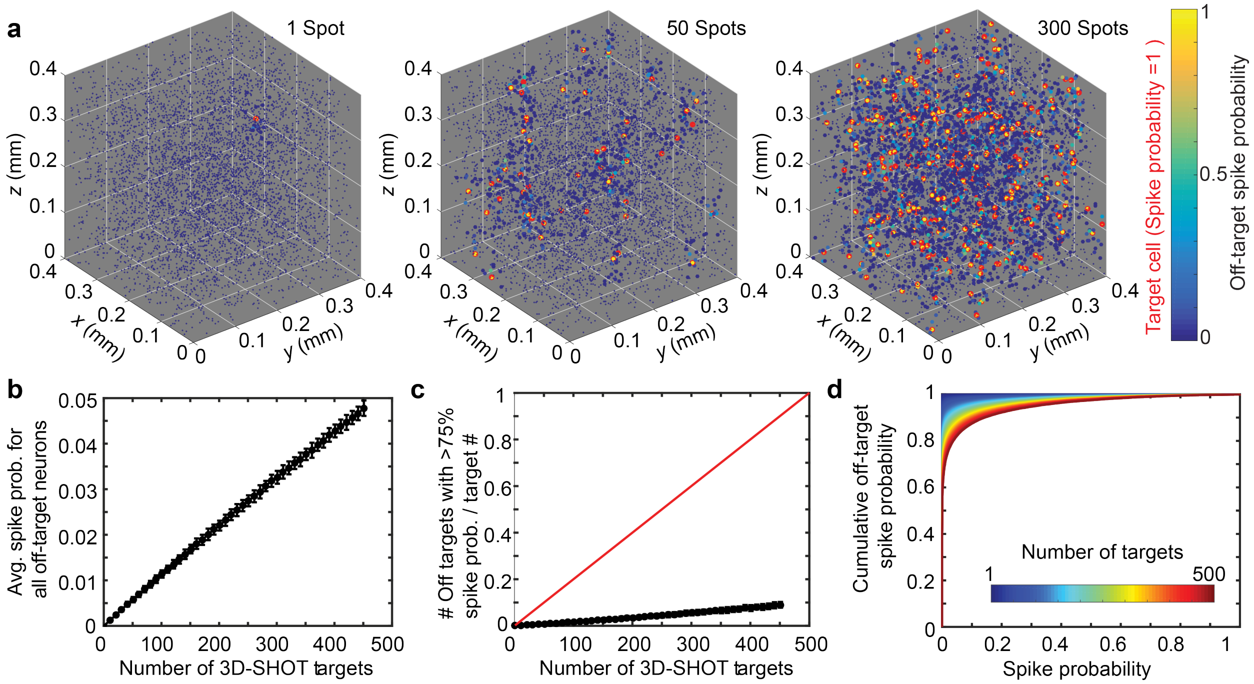

Supplementary Figure 11: **Photostimulation in simulated brain tissue.** (a) Example resolution simulations showing volumes of simulated brain tissue with 1, 50, or 300 3D-SHOT spots. Neurons that are stimulation targets are shown in red, and off-target neurons are color coded by spike probability. (b) Average of the spike probability of all off-target neurons as a function of 3D-SHOT target number (mean and standard deviation of 100 random sets of targets). (c) Ratio of the number of reliably driven off-target neurons ($> 75\%$ spike probability) as a function of increasing numbers of 3D-SHOT spot targets (data represent mean and standard deviation of 100 random target sets). (d) Cumulative probability of estimated spike probability of off-target neurons for increasing numbers of 3D-SHOT target numbers (see colorbar). Data are the concatenations of 100 trials.

FWHM	$\boldsymbol{E}$-field	Intensity $I = \|\boldsymbol{E}\|^2$	2-photon absorption I^2
in B	$2\sqrt{ln(2)}s$	$\sqrt{2ln(2)}s$	$\sqrt{ln(2)}s$
in C	$\frac{f_2}{f_1}2\sqrt{ln(2)}s$	$\frac{f_2}{f_1}\sqrt{2ln(2)}s$	$\frac{f_2}{f_1}\sqrt{ln(2)}s$
in E	$\frac{f_2 f_5}{f_1 f_4}2\sqrt{ln(2)}s$	$\frac{f_2 f_5}{f_1 f_4}\sqrt{2ln(2)}s$	$\frac{f_2 f_5}{f_1 f_4}\sqrt{ln(2)}s$
in F	$\frac{f_2 f_5 f_7}{f_1 f_4 f_6}2\sqrt{ln(2)}s$	$\frac{f_2 f_5 f_7}{f_1 f_4 f_6}\sqrt{2ln(2)}s$	$\frac{f_2 f_5 f_7}{f_1 f_4 f_6}\sqrt{ln(2)}s$
FWHM : Setup 1	$\boldsymbol{E}$-field	Intensity $I = \|\boldsymbol{E}\|^2$	2-photon absorption I^2
in B	4.0 mm	2.8 mm	2.0 mm
in C	$1.3 * 10^3$ µm	$9.3 * 10^2$ µm	$6.6 * 10^2$ µm
in E	$7.9 * 10^2$ µm	$5.6 * 10^2$ µm	$4.0 * 10^2$ µm
in F	20 µm	14 µm	9.9 µm
FWHM - Setup 2	$\boldsymbol{E}$-field	Intensity $I = \|\boldsymbol{E}\|^2$	2-photon absorption I^2
in B	5.0 mm	3.5 mm	2.5 mm
in C	$9.9 * 10^2$ µm	$7.0 * 10^2$ µm	$5.0 * 10^2$ µm
in E	$4.0 * 10^2$ µm	$2.8 * 10^2$ µm	$2.0 * 10^2$ µm
in F	20 µm	14 µm	9.9 µm

Supplementary Table 1: **Conversion table for calibration of the CTFP dimensions.** We show the dimensions (Full Width Half Max) for the Gaussian beam at selected focal planes : B, C, E, and F. Characteristic dimensions are shown for the electric field, the Intensity, and for two-photon absorption. Experimental values are shown for Setup 1, and Setup 2. In both cases, the CTFP is engineered for a 10 µm diameter temporally focused disk area target in F for two-photon neural photostimulation.

Variable	Description	Setup 1	Setup 2
λ_0	Laser wavelength	1040 nm	1030 nm
τ	Laser pulse duration (FWHM =$2\sqrt{2ln(2)}\tau$)	63 fs	117 fs
s	Gaussian beam diameter (FWHM = $s\sqrt{ln(2)}$)	2.4 mm	2.9 mm
d	Gaussian beam phase sphericity at grating	$\sqrt{\lambda_0 f_c}$	$\sqrt{\lambda_0 f_c}$
γ	Grating spectral response coefficient	$\gamma = \lambda_0/2\pi ch$	$\gamma = \lambda_0/2\pi ch$
h	Blazed grating frequency	300 mm^{-1}	600 mm^{-1}
f_1	Focal length (demagnifier)	60 mm	100 mm
f_2	Focal length (demagnifier)	20 mm	20 mm
f_3	Focal length (beam expansion)	200 mm	200 mm
f_4	Focal length (beam expansion)	500 mm	500 mm
f_5	Focal length (Fourier hologram lens)	300 mm	200 mm
f_6	Focal length (tube lens)	200 mm	200 mm
f_7	Focal length (40×, resp 20× objective)	5 mm	10 mm
f_c	Focal length (for spherical phase curvature)	1000 mm	1000 mm

Supplementary Table 2: **Experimental setup properties for 3D-SHOT.** To design a 10 μm excitation disk with the CTFP, we have implemented 3D-SHOT on two separate systems. The reference system is Setup 1, except for Figure 4 and Figure 5, which show results obtained with Setup 2.

Supplementary Note 1. Time-bandwidth limited femtosecond Gaussian pulses.

In the following supplementary notes, we consider the isometric definition of the Fourier Transform $\mathcal{F}_{u,v} : f \to \hat{f}$ and its inverse given by :

$$\hat{f}(v) = \frac{1}{\sqrt{2\pi}} \int_{-\infty}^{\infty} f(u) e^{iuv} \mathrm{d}u \qquad f(u) = \frac{1}{\sqrt{2\pi}} \int_{-\infty}^{\infty} \hat{f}(v) e^{-iuv} \mathrm{d}v \tag{1}$$

The optical path is represented in Supplementary Figure 1. For two-photon photo-excitation in brain tissue the light source is a femtosecond laser with a central wavelength of $\lambda_0 = 1040$nm. At 80MHz repetition rate or below, pulses can be treated independently. In position A, we model the complex amplitude of a single time-bandwidth limited pulse in time and space by:

$$A_{\mathrm{A}}(x, y, \Delta t) = \frac{2^{1/4}}{\pi^{3/4} s \sqrt{\tau}} e^{-\frac{x^2+y^2}{s^2}} e^{-\frac{\Delta t^2}{4\tau^2}} e^{ic\Delta t/\lambda_0} \tag{2}$$

where s is the spatial width of the Gaussian pulse at the system input, and τ the pulse duration. Our model assumes normalized energy in a given laser pulse :

$$\int_{\mathbb{R}^3} |A_\text{A}|^2(x, y, \Delta t)\, dx\, dy\, \mathrm{d}\Delta t = 1 \tag{3}$$

In the spectral domain, $\Delta\omega = c\Delta k$, and we can rewrite the amplitude as:

$$A_\text{A}(x, y, \Delta\omega) = \mathcal{F}_{\Delta t, \Delta\omega}\left[A_\text{A}\right](x, y, c\Delta k) = \frac{2^{3/4}\sqrt{\tau}}{s\pi^{3/4}} e^{-(c\Delta k\tau)^2} e^{-\frac{x^2+y^2}{s^2}} \tag{4}$$

A pulse compressor can be used to selectively delay the spectral components of pulses to compensate for unwanted dispersion along the optical path and to ensure that the perceived pulses at the desired temporally focused pulses are time-bandwidth limited.

Supplementary Note 2. Computer Generated Holography (CGH) with phase modulation in Fourier space.

System Point Spread Function (PSF) for Computer Generated Holography (CGH). The experimental setup (Supplementary Figure 1) is designed to compare the respective performance of our method and conventional holography. For this, two movable mirrors on a translation stage enable easy switching between two different light paths, both with the same phase-only SLM in Fourier space. Here, we first consider the optical path for conventional 3D holography (in red). In position C, the amplitude is given by:

$$A_\text{C}(x, y, \Delta k) = \mathcal{F}_{(x,y),(k_x,k_y)}\left[A_\text{A}\right]\left(\frac{x}{\lambda_0 f_3}, \frac{y}{\lambda_0 f_3}, \Delta k\right) \tag{5}$$

$$A_\text{C}(x, y, \Delta k) = \frac{s\sqrt{\tau}}{2^{1/4}\pi^{3/4} f_3 \lambda_0} e^{-c^2\Delta \text{k}^2\tau^2 - \frac{1}{4}s^2\left(\frac{x^2}{f_3^2\lambda_0^2} + \frac{y^2}{f_3^2\lambda_0^2}\right)} \tag{6}$$

At the input face of the SLM, in position D^-, the field becomes a magnified, inverted, copy of the field in A, and the magnification is given by $M_{3,4} = -f_3/f_4$.

$$A_\text{D}^-(x, y, \Delta k) = \mathcal{F}_{(x,y),(k_x,k_y)}\left[A_\text{C}\right]\left(\frac{x}{\lambda_0 f_4}, \frac{y}{\lambda_0 f_4}, \Delta k\right) \tag{7}$$

$$A_\text{D}^-(x, y, \Delta k) = M_{3,4} A_\text{A}(M_{3,4}x, M_{3,4}y, \Delta k) \tag{8}$$

At the output face of the SLM, the beam in position D^+ becomes:

$$A_\text{D}^+(x, y, \Delta k) = A_\text{D}^-(x, y, \Delta k)\mathcal{H}(x, y) \tag{9}$$

where $\mathcal{H}(x, y) = e^{i\phi(x,y)}$ is the digital phase mask applied onto the SLM. Femtosecond pulses have a fairly narrow bandwidth ($\approx$ 3nm) and SLM-induced dispersion is not an issue for holography.

$$A_\text{E}(x, y, \Delta k) = \mathcal{F}_{(x,y),(k_x,k_y)}\left[A_\text{D}^+\right]\left(\frac{x}{\lambda_0 f_5}, \frac{y}{\lambda_0 f_5}, \Delta k\right) \tag{10}$$

$$A_\text{E}(x, y, \Delta k) = \mathcal{F}_{(x,y),(k_x,k_y)}\left[A_\text{D}^+\right](\frac{x}{\lambda_0 f_5}, \frac{y}{\lambda_0 f_5}, \Delta k) \otimes \mathcal{F}_{(x,y),(k_x,k_y)}\left[\mathcal{H}\right](\frac{x}{\lambda_0 f_5}, \frac{y}{\lambda_0 f_5}) \tag{11}$$

$$A_\text{E}(x, y, \Delta k) = \text{PSF}_\text{E}(x, y, \Delta k) \otimes \hat{\mathcal{H}}(\frac{x}{\lambda_0 f_5}, \frac{y}{\lambda_0 f_5}) \tag{12}$$

where $\otimes$ represents the convolution product and,

$$\mathrm{PSF_E}(x,y,\Delta k) = \frac{s\sqrt{\tau}}{2^{1/4}\pi^{3/4}f_5\lambda_0 M_{3,4}} e^{-c^2\Delta k^2\tau^2 - \frac{s^2\left(x^2+y^2\right)}{4f_5^2\lambda_0^2 M_{3,4}^2}} \tag{13}$$

is the normalized Point Spread Function (PSF) of the optical system, at the focal plane in E. Finally, the tube lens L_6 and objective, L_7 further demagnify the synthesized wave-front, and the resulting holographic pattern at the focal plane of the objective F is given by :

$$A_\mathrm{F}(x,y,\Delta k) = M_{6,7}\mathrm{PSF_E}(M_{6,7}x, M_{6,7}y, \Delta k) \otimes \hat{\mathcal{H}}(\frac{xM_{6,7}}{\lambda_0 f_5}, \frac{yM_{6,7}}{\lambda_0 f_5}) \tag{14}$$

where with $M_{6,7} = -f_6/f_7$ is the magnification of the microscope assembly.

Supplementary Note 3. 3D Scanless Holographic Optogenetics with Temporal focusing.

Space, phase, and spectral shaping of femtosecond pulses. We now consider the secondary path (Supplementary Figure 1, blue path) for 3D-SHOT. We apply a spherical phase pattern to the laser beam using lens L_C to adjust the size, s, and the sphericity, d, of the field before frequency-specific diffraction on the blazed grating. The distance between lens L_C and the diffraction grating is effectively reduced to zero using a set of 200mm lenses in a 4-f configuration which places a virtual image of the lens directly onto the diffraction grating. In location B, the field just before reflection on the diffraction grating is therefore given by the time-dependent amplitude A_B :

$$A_\mathrm{B}^-(x,y,\Delta k) = A_\mathrm{A}(x,y,\Delta k)e^{i\frac{x^2+y^2}{d^2}} \tag{15}$$

The grating implements a wavelength dependent phase-shift given by : $e^{i\gamma c\Delta kx}$, where γ represents the wavelength-specific response of the grating (Supplementary Table 1), and Δk represents the variation of the wavevector distribution around the central wavelength, $\lambda_0 = 1040\mathrm{nm}$, of the laser. The actual wavelength to be considered within the bandwidth of the pulse is given by : $\lambda = \lambda_0 + \Delta\lambda$.

$$\Delta\lambda = \frac{\lambda_0^2}{2\pi}\Delta k\, x \tag{16}$$

At the output face of the grating, the complex amplitude in the first diffracted order is given by :

$$A_\mathrm{B}^+(x,y,\Delta k) = A_\mathrm{B}^-(x,y,\Delta k)e^{i\gamma c\Delta kx} \tag{17}$$

We use a blazed grating optimized for diffraction in the near-infrared range (1000nm blaze depth). Lens L_1 and L_2 are placed in a 4-f configuration, with $M_{1,2} = -f_1/f_2$. A large demagnification has the advantage of allowing high average power levels (up to 18W average power tested) without damaging the diffraction grating. The amplitude in location C is given by :

$$A_\mathrm{C}(x,y,\Delta k) = \left(\frac{2}{\pi}\right)^{3/4} \frac{M_{1,2}\sqrt{\tau}}{s} e^{-c^2\Delta \mathrm{k}^2\tau^2 - ic\gamma\Delta\mathrm{k}M_{1,2}x + \frac{iM_{1,2}^2\left(x^2+y^2\right)}{d^2} - \frac{M_{1,2}^2\left(x^2+y^2\right)}{s^2}} \tag{18}$$

In the SLM plane D, the amplitude is given by :

$$A_{\mathrm{D}}(x,y,\Delta k) = \mathcal{F}_{(x,y),(k_x,k_y)}\left[A_{\mathrm{C}}\right]\left(\frac{x}{\lambda_0 f_4}, \frac{y}{\lambda_0 f_4}, \Delta k\right) \tag{19}$$

$$A_{\mathrm{D}}(x,y,\Delta k) = \frac{d^2 s\sqrt{\tau}}{\sqrt[4]{2}\pi^{3/4} f_4 \lambda_0 M_{1,2}\left(d^2 - is^2\right)} e^{-c^2\Delta k^2\tau^2 - \frac{d^2 s^2(-c\gamma\Delta k f_4\lambda_0 M_{1,2}+x-iy)(-c\gamma\Delta k f_4\lambda_0 M_{1,2}+x+iy)}{4f_4^2\lambda_0^2 M_{1,2}^2(d^2-is^2)}} \tag{20}$$

We display $|A_{\mathrm{D}}(x,y,\Delta k)|^2$ for various values of $\Delta\lambda = 2\pi/\Delta k$ (Supplementary Figure 2a). Summing across the frequency spectrum, the intensity on the SLM, in D (Supplementary Figure 2b) becomes :

$$I_{\mathrm{D}}(x,y) = \int |A_{\mathrm{D}}(x,y,\Delta k)|^2 \mathrm{d}\Delta k = \frac{d^4 s^2 \tau e^{-\frac{d^4 s^2\left(d^4\left(\gamma^2 s^2 y^2+4\tau^2\left(x^2+y^2\right)\right)+4s^4\tau^2\left(x^2+y^2\right)\right)}{2f_4^2\lambda_0^2 M_{1,2}^2(d^4+s^4)(d^4(\gamma^2 s^2+4\tau^2)+4s^4\tau^2)}}}{2\pi c f_4^2 \lambda_0^2 M_{1,2}^2\sqrt{\left(d^4+s^4\right)\left(d^4\left(\gamma^2 s^2+4\tau^2\right)+4s^4\tau^2\right)}} \tag{21}$$

The beam size, s, determines the dimensions of the temporally focused disk image at the focal plane of the objective. The ability to spatially distribute the hologram within the volume of interest relies on efficient diffraction of coherent light by the SLM. This requires that each wavelength within the pulse spectrum should be spatially distributed on a significant effective area of the SLM to maximize the number of pixels contributing to the hologram. For this purpose, the sphericity of the input beam, d, is a degree of freedom independent from the beam size, which we can adjust to control how light is spatially distributed in the Fourier domain.

This fundamental aspect of our method is illustrated in Supplementary Figure 2. In the absence of phase patterning of the incoming beam, for instance by removing lens $\mathrm{L_C}$, the amplitude in D simplifies to $A^{\star}_{\mathrm{D}}$:

$$\lim_{d\to+\infty} A^{\star}_{\mathrm{D}}(x,y,\Delta k) = \frac{s\sqrt{\tau}}{\sqrt[4]{2}\pi^{3/4} f_4\lambda_0 M_{1,2}} e^{-c^2\Delta k^2\tau^2 - \frac{s^2\left((x-c\gamma\Delta k f_4\lambda_0 M_{1,2})^2+y^2\right)}{4f_4^2\lambda_0^2 M_{1,2}^2}} \tag{22}$$

and is shown in Supplementary Figure 2c. The corresponding intensity on the SLM (Supplementary Figure2d) becomes :

$$I^{\star}_{\mathrm{D}}(x,y) = \frac{\sqrt{2}s^2\tau}{\pi^{3/2} f_4^2\lambda_0^2 M_{1,2}^2\left(\gamma^2 s^2+4\tau^2\right)} e^{-\frac{4\Delta t^2 f_4^2\lambda_0^2 M_{1,2}^2+\gamma^2 s^4 y^2+4s^2\tau^2\left(x^2+y^2\right)}{2f_4^2\lambda_0^2 M_{1,2}^2(\gamma^2 s^2+4\tau^2)}} \tag{23}$$

Not applying a well calibrated phase mask to the laser beam results in the effective coverage of the SLM being reduced to a diffraction limited spot, with a wavelength-specific displacement along the direction of the diffraction grating, as illustrated in Supplementary Figure 2c,d. In this case the SLM would be partially illuminated with a line object, and each portion of the spectrum would span across a small (≈ 30) number of pixels. Besides potential damage to the SLM, diffraction efficiency is reduced to a minimum, and the corresponding temporally focused image at the objective cannot be efficiently focused at desired locations within the 3D imaging volume by the SLM.

To address this potential issue and enable holographic replication of the CTFP at custom 3D locations while keeping the SLM in Fourier space, a well calibrated phase pattern needs to be applied to the beam just before reflection on the diffraction grating (here the phase mask and the diffraction grating are virtually superimposed with relay lenses). Many types of phase masks can be considered to ensure proper coverage of the SLM throughout the pulse spectrum. Here, we choose a simple lens L_C. This strategy is not only simple, but also has the additional advantage to provide an analytical expression of the electric field in space and time throughout the optical setup. The spectral distribution of light on the SLM is shown in Supplementary Figure 2a. The ability of the SLM to diffract light for holography is restored to similar diffraction efficiency levels as in conventional holography methods, and the accessible volume compares to conventional CGH.

$$A_{\mathrm{E}}(x, y, \Delta k) = \mathcal{F}_{(x,y),(k_x,k_y)}\left[A_{\mathrm{D}}^{+}\right]\left(\frac{x}{\lambda_0 f_5}, \frac{y}{\lambda_0 f_5}, \Delta k\right) \tag{24}$$

$$A_{\mathrm{E}}(x, y, \Delta k) = \mathrm{CTFP}_{\mathrm{E}}(x, y, \Delta k) \otimes \hat{\mathcal{H}}\left(\frac{x}{\lambda_0 f_5}, \frac{y}{\lambda_0 f_5}\right) \tag{25}$$

where $\otimes$ represents the convolution product. $\mathrm{CTFP}_{\mathrm{E}}(x, y)$ represents the complex valued, normalized, **C**ustom-made **T**emporally **F**ocused **P**attern of the optical system, at the focal plane in F.

$$\mathrm{CTFP}_{\mathrm{E}}(x, y, \Delta k) = \frac{\left(\frac{2}{\pi}\right)^{3/4} M_{1,5}\sqrt{\tau}}{s} e^{-c^2\Delta \mathrm{k}^2\tau^2 + ic\gamma\Delta \mathrm{k} M_{1,5}x + \frac{iM_{1,5}^2\left(x^2+y^2\right)}{d^2} - \frac{M_{1,5}^2\left(x^2+y^2\right)}{s^2}} \tag{26}$$

where $M_{1,5} = \frac{f_1}{f_2}\frac{f_4}{f_5}$. The microscope tube lens and objective further demagnify the signal by a factor f_6/f_7, and the resulting CTFP, designed to match the characteristic size of a neuron for photostimulation applications, is given by :

$$\mathrm{CTFP}(x, y, \Delta k) = \frac{\left(\frac{2}{\pi}\right)^{3/4} M_{1,7}\sqrt{\tau}}{s} e^{-c^2\Delta \mathrm{k}^2\tau^2 + ic\gamma\Delta \mathrm{k} M_{1,7}x + \frac{iM_{1,7}^2\left(x^2+y^2\right)}{d^2} - \frac{M_{1,7}^2\left(x^2+y^2\right)}{s^2}} \tag{27}$$

where $M_{1,7} = -\frac{f_1}{f_2}\frac{f_4}{f_5}\frac{f_6}{f_7}$.

Custom-made Temporally Focused Pattern (CTFP). Assuming no phase pattern is present on the SLM, $A_{\mathrm{F}}(x, y, \Delta k) = \mathrm{CTFP}(x, y, \Delta k)$, the intensity is given by :

$$I_{\mathrm{F}}(x, y, \Delta t) = \left|\mathcal{F}_{\Delta\omega,\Delta t}\left[A_{\mathrm{F}}(x, y, \Delta\omega/c)\right]\right|^2 \tag{28}$$

$$I_{\mathrm{F}}(x, y, \Delta t) = \frac{\sqrt{2}M_{1,7}^2}{\pi^{3/2}s^2\tau} e^{-\frac{2M_{1,7}^2\left(x^2+y^2\right)}{s^2} - \frac{(\Delta \mathrm{t} + \gamma M_{1,7}x)^2}{2\tau^2}} \tag{29}$$

and the time-averaged two-photon absorption profile in F is given by :

$$I_{\mathrm{F}}^2(x, y) = \int I_{\mathrm{F}}^2(x, y, \Delta t)\mathrm{d}\Delta t = \left(\frac{2M_{1,7}^2}{\pi s^2} e^{-\frac{2M_{1,7}^2\left(x^2+y^2\right)}{s^2}}\right)^2 \tag{30}$$

To compute the CTFP in 3D, we use Fresnel propagation and we define :

$$\tilde{A}_{\mathrm{F}}(k_x, k_y, \Delta k) = \mathcal{F}_{(x,y),(k_x,k_y)}\left[A_{\mathrm{F}}\right] \tag{31}$$

$$\mathcal{P}(k_x, k_y) = \mathcal{F}_{(x,y),(k_x,k_y)} \left[e^{\frac{i\pi x^2}{\lambda_0 z}} e^{\frac{i\pi y^2}{\lambda_0 z}} \right] = ie^{-\frac{i\lambda_0 z\left(k_x^2+k_y^2\right)}{4\pi}} \tag{32}$$

The defocused field is given by propagating each wavelength independently :

$$A_{\mathrm{F}}(x, y, z, \Delta k) = \mathcal{F}^{-1}_{(k_x,k_y),(x,y)} \left[\mathcal{P}(k_x, k_y) A_{\mathrm{F}}(k_x, k_y, \Delta k)\right] \tag{33}$$

Which can be then expressed as a function of time :

$$A_{\mathrm{F}}(x, y, z, \Delta t) = \mathcal{F}_{\Delta\omega,\Delta t} \left[A_{\mathrm{F}}(x, y, z, \Delta\omega/c)\right] \tag{34}$$

$$A_{\mathrm{F}}(x, y, z, \Delta t) = K_0 e^{\frac{-i\pi M_{1,7}^2\left(d^2-is^2\right)\left(x^2+y^2\right)\left(d^2\left(\lambda_0 M_{1,7}^2 z\left(\gamma^2 s^2+4\tau^2\right)-4i\pi s^2\tau^2\right)-4i\lambda_0 M_{1,7}^2 s^2\tau^2 z\right)}{\left(\lambda_0 M_{1,7}^2 s^2 z+d^2\left(\pi s^2+i\lambda_0 M_{1,7}^2 z\right)\right)\left(4\lambda_0 M_{1,7}^2 s^2\tau^2 z+d^2\left(4\pi s^2\tau^2+i\lambda_0 M_{1,7}^2 z(\gamma^2 s^2+4\tau^2)\right)\right)}} \times e^{\frac{\left(d^2\left(\Delta t\lambda_0 M_{1,7}^2 z+i\pi\gamma M_{1,7} s^2 x-i\pi\Delta t s^2\right)-i\Delta t\lambda_0 M_{1,7}^2 s^2 z\right)^2}{\left(\lambda_0 M_{1,7}^2 s^2 z+d^2\left(\pi s^2+i\lambda_0 M_{1,7}^2 z\right)\right)\left(4\lambda_0 M_{1,7}^2 s^2\tau^2 z+d^2\left(4\pi s^2\tau^2+i\lambda_0 M_{1,7}^2 z(\gamma^2 s^2+4\tau^2)\right)\right)}} \tag{35}$$

where K_0 is the normalization constant.

Simulation results are shown in Supplementary Figure 3. The CTFP shows temporal focusing at $z = 0$ µm, a virtual image of the diffraction grating where simultaneous constructive interference of multiple-wavelength specific light paths (Supplementary Figure 3a) re-creates a femtosecond pulse image and yields the strong nonlinear response needed for two-photon response(Supplementary Figure 3b). At the temporal scale of a single pulse, the temporal focus is a line object along the $\boldsymbol{y}$ axis swept along the $\boldsymbol{x}$ axis. Additionally, a secondary focus at $z = 25$ µm corresponds to a geometric focus (from lens L_C) which is different for each wavelength in the pulse bandwidth. At any point in space along the secondary focus line, the perceived pulse is narrow in the frequency domain, and therefore broadened in time (Supplementary Figure 3c), which reduces two-photon absorption (Supplementary Figure 3d).

For 3D-SHOT simulation (Fig. 2d), we computed the evolution of pre-shaped wave-packets in time and space step-by-step through the optical system (Supplementary Notes 1-3). Simulation closely matches the measured fluorescent response for 3D-SHOT. Simulation also provides space-time projections (Fig. 2e) showing the fundamental difference between the primary and the secondary focus (Fig. 1, green and pink dashed line, respectively). The primary focus (green arrow) is a virtual image of the diffraction grating and shows constructive interference that locally reconstructs the temporally focused beam. The phase-shaping lens, Lc, introduces a secondary geometrically focused line image 25 m above the primary focus (pink arrow). Space-time projections from simulation indicate that the secondary focus corresponds to a spectral decomposition of the femtosecond pulse, and is not temporally focused. Since two-photon photo-activation is proportional to the time-averaged square of the light intensity, the amount of photostimulation in the secondary focus is proportional to the inverse of the perceived pulse duration. The secondary focus benefits from attenuation from temporal stretching despite being more spatially confined than the primary focus, and therefore does not significantly affect spatial resolution, as shown experimentally and in simulation.

The experimental setup is designed for specific CTFP dimensions designed by the user. Supplementary Table 1 summarizes the scaling of the CTFP throughout the system at each repetition of

the temporally focused plane. The SLM is a Hamamatsu LCOS (X10468-03) with 600×800 pixels (20 µm pixel size). In setup 1, the objective is a water immersion, $40\times$, NA= 0.8 objective, and the laser is a fidelity HP18, providing 150 fs pulses with 18W average power at a repetition rate of 80MHz. Setup 2 uses an Amplitude Satsuma HP2 laser providing 280fs pulses with 20W average power at 1MHz, and a water immersion, 20X, NA= 1.0 microscope objective. By design, setup 2 enables a larger volume of operation with similar CTFP dimensions. All lenses are achromatic doublets with anti-reflection coating for near-IR applications. The Group Velocity Dispersion (GVD) can be compensated in the laser unit by applying a spectral chirp to the laser beam that minimizes pulse duration at the objective. To facilitate the replication of our system, the experimental setup properties are summarized in Supplementary Table 2.

Supplementary Note 4. Accessible volume

The area of the experimental setup that synthesizes holograms is shown in Supplementary Figure 4. By definition, the microscopes magnification, M, is given by:$M = \frac{f_6}{f_7}$. To optimize the resolution of the microscope, the SLM image is matched to the dimensions of the back aperture: $\frac{p}{d} = \frac{f_5}{f_6}$.

The accessible range of the hologram at the objective is given by considering the maximal accessible angle for a first order diffraction (one wavelength phase shift over the size of one pixel). We deduce the accessible range in the image (x,y) plane:

$$\Delta_{xy} = \frac{\lambda_0 f_5}{Mp}, \tag{36}$$

and the accessible range along the optical (z) axis:

$$\Delta_z = \frac{\lambda_0 f_5^2}{M^2 ph}, \tag{37}$$

with the wavelength of the laser $\lambda_0 = 1030\text{nm}$. With a 20X microscope objective, the theoretical limits of the accessible volume in our system (Setup 2, Supplementary Table 1) are: $\Delta_{xy} = 500$ µm and $\Delta_z = 650$ µm. We note that the accessible range can be improved by reducing the pixel size p. SLMs with smaller pixels are commercially available, but resolution comes at the expense of diffraction efficiency. Here, we have purposefully selected an SLM with pixels to minimize photon losses in high power applications. For high resolution SLMs with smaller pixel size, new limiting factors such as the diameter of the tube lens, and the microscopes ability to accept high incidence angles, should be taken into account in determining the accessible volume for photostimulation purposes. In practice, the accessible volume and spatial resolution can be improved by considering larger SLMs with higher pixel count, and performance can be improved if desired up until the microscope objective becomes again the limiting factor for resolution and imaging volume.

Supplementary Note 5. Optimizing two-photon intensity levels for photostimulation.

Selecting the appropriate amount of power for neural photostimulation relates both to the spatial distribution of two photon absorption and cell physiology. For two photon absorption, we

consider a simple Gaussian response model for photostimulation with the CTFP with FWHM= $2\sqrt{2ln(2)}\sigma \approx 30$ µm (Supplementary Figure 5a). The photocurrent, J, is shown for various levels of normalized photostimulation light intensity, I_0, and is given, in first approximation, by:

$$J(z) = I_0^2 e^{-z^2/2\sigma^2} \tag{38}$$

This corresponds for instance to the spatial distributions of photocurrents observed in each peak of the two-target experiment (Fig. 6b,c). Here, we consider a simple threshold model for which the spike probability, P, is given by a sigmoidal response profile:

$$P = \frac{1}{1 + e^{-(J-J_t)/\Delta J}} \tag{39}$$

where J_t is the average threshold photocurrent required to trigger an action-potential, and ΔJ determines the spiking probability of neurons near threshold value (Supplementary Figure 5b). Combining Equations 38 and 39 yields the spiking probability distribution as a function of light intensity (Supplementary Figure 5c) and shows three characteristic regimes. The first case, $I_0^2 < J_t - \Delta J$ (I), represents a below-threshold photostimulation for which action potentials are unlikely to be triggered. For $J_t - \Delta J < I_0^2 < J_t + \Delta J$ (II), the amount of power received by the neuron is near the threshold value and action-potential are likely be triggered. For intensities above this range (III), the probability of spiking the cell is almost certain, but the FWHM gradually increases as photocurrents induced by defocused light remain intense enough to permit action-potentials (Supplementary Figure 5d). Near the optimal value, we observe that the nonlinearity of action potential response has the advantage of further narrowing the FWHM. For instance, a 30 µm FWHM for photocurrent can yield a 20 µm FWHM for spiking probability.

To quantify the degree of photostimulation cross-talk, we defined the spatial contrast coefficient $\gamma = I_0/(I_1 + I_2)$ where I_1 and I_2 are the photocurrents at either peak corresponding to each target, and I_0 is the photocurrent recorded when the cell is exactly between targets. With conventional holography, $\gamma = 3.2 * 10^{-1}$ in CHO cells (Fig. 6b), and $\gamma = 6.9 * 10^{-1}$ in mouse brain slice (Fig. 6c). With 3D-SHOT, $\gamma = 1.3 * 10^{-5}$ in CHO cells (Figure 6b), and $\gamma = 1.9 * 10^{-2}$ in mouse brain slice (Fig. 6c) . When considering spiking probability in neurons, we observed $\gamma = 0$ with 3D-SHOT (Fig. 6d) indicating that a neuron surrounded by two targets can be successfully protected from undesired photo-activation. (Supplementary Note 5)

Depending on the end-user preference, this model shows how different power levels can be considered to either guarantee single cell resolution at the risk of affecting the spiking probability (II), or to ensure the photo-activation of a given neuron and triggering at least one action potential on demand at the risk of stimulating other cells in the vicinity of the target (III). Since 3D-SHOT offers the narrowest possible depth selectivity for inducing photocurrents in any set of neurons in a large volume, the necessary trade-off between spike probability and spatial resolution is not incompatible with offering both single cell spatial resolution and enabling all-optical triggering of action potentials with a high probability of success.

Supplementary Note 6. Spatial power calibration

To activate n neurons located at positions $\boldsymbol{r}_i$ within the previously described accessible volume, our goal is to compute a hologram and select the appropriate amount of laser intensity, I , that will achieve a custom distribution of intensity $I_{i,i=1...n}$ in selected neurons given by while minimizing the amount of light received by other neurons within the volume of interest.

In general, $I > \sum_{i=1}^{n} I_i$, since a fraction of the photons emitted by the laser will not reach any of the targets. There are two mechanisms responsible for inefficiency in the system. First, physical-losses along the optical path that relate to reflections at optical interfaces, limited numerical aperture, or absorption, which are common to any optical system. Second, the SLM introduces additional holographic losses which correspond to photons that are not focused onto any of the desired targets, but wrongly deflected by the SLM either somewhere else in the volume of interest or outside the optical system in higher order interferences. Such losses are specific to the pattern placed on the SLM (finite pixel size, discrete dynamic range, electronic noise and phase-only control of the wavefronts).

Both sources of photon loss need to be fully characterized to compute holograms that accurately provide the desired distribution of light intensity on each targeted neuron. Physical losses are specific to each targeted location and can be accounted for and compensated with a simple 3D calibration. However, a power calibration is not sufficient to address both categories, because holographic losses related to the SLMs response depend on the respective 3D location of all targets.

Since holographic losses are unique to each point cloud distribution, we have developed a simulation tool to quantify them. We simulate the volume distribution of intensity for any given phase mask on the SLM. This simulation is quantitative and provides a reliable estimate of the expected intensity profile at any depth under the microscope objective (neglecting for system-related losses). The simulation allows us to estimate what fraction of a unitary amount of light intensity, μ_i , would reach each target (Supplementary Figure 6a) in a perfect optical system.

To quantify the physical losses induced by unavoidable imperfections of the optical elements along the light path, we proceed to a spatial calibration. For this, we compute $N = 15000$ calibration holograms that each target one random location within the volume of interest, and we use the previous simulation to estimate the holographic diffraction efficiency (DE) for each hologram. We then set the laser power to a reference intensity level, I , and we measure the collected intensity under the microscope objective, I_i , either with a photodiode, or by recording the two-photon response in a thick slide of fluorescent material (Supplementary Figure 6b). The physical loss, p_i, for the optical system at the location is therefore given by:

$$p_i(r_i) = \frac{I_i}{I\mu_i} \tag{40}$$

Calibration results are shown in Supplementary Figure 6c. We then estimate physical losses at any location within the volume of interest by direct interpolation on calibration data points.

Lets now consider a set of n neurons located at positions $\boldsymbol{r}_i$. To compute a holographic pattern that will illuminate each target with intensity I_i, we start by estimating the amount of attenuation induced by the optical system, p_i. This step is made possible by spatial interpolation of data points acquired during the power calibration (Supplementary Figure 6c). We then compute a hologram for a point cloud at positions for which we specify the desired power distribution given by: I_i/p_i.

The hologram computation yields the phase mask to be displayed onto the SLM, as well as a total diffraction efficiency (DE) that corresponds to the fractional amount of incoming photons that will reach at least one of the desired targets. The laser power required to illuminate the targets with the desired intensity level is therefore given by:

$$I = \frac{1}{\text{DE}} \sum_{i=1}^{n} \frac{I_i}{p_i(\boldsymbol{r}_i)} \tag{41}$$

Due to variations in intrinsic excitability and opsin expression levels, different neurons will require different amounts of light to be photo-activated. In practice, the proposed method can tailor the excitation energy directed into each spot to match the needs of each targeted neuron. This capability is critical especially in large scale excitation experiments with scanless holographic systems operating near the range limits of their microscope objective. Finally, combining the spatial power calibration and hologram simulation provides a simple feasibility criteria for each photostimulation pattern by indicating directly if the desired power distribution in a given set of simultaneous targets can be physically achieved with the available laser power.

Supplementary Note 7. 3D-SHOT performance with multi-target holograms through brain tissue.

We recorded two-photon absorption for various holograms through mouse brain slices of increasing thickness (Supplementary Figure 7a), ranging from 0 to 800 µm, with various locations in space and typical separation distance (Supplementary Figure 7b).

Our findings is that 3D-SHOT spatial resolution through brain tissue performs equally or better than similar technologies using two-photon photostimulation and temporal focusing. The FWHM of the effective targeted area degrades along the radial (Supplementary Figure 7c) and axial (Supplementary Figure 7d) directions in deeper layers of the brain. In addition to gradual losses of spatial resolution near the targeted location, intense scattered light in deeper layers of brain tissue reduce contrast and create a non-zero speckled background illumination pattern near non-targeted locations within the volume of interest. The system eventually fails as non targeted neurons receive significant amounts of light leading to inadvertent photostimulation. We computed the total amount of two-photon absorption induced in the non-targeted volume (Supplementary Figure 7e), as a function of brain tissue thickness, and we deduce that a depth through brain tissue of 500 µm represents a practical operational limit for spatial resolution.

Statistical data is shown in Supplementary Figure 8. For each target and for each hologram, the

dimensions of the targeted volume were measured and recorded along with relevant quantities such as the target location in space, the target distance to the nearest target, and the number of targeted locations in the volume being recorded. Using principal component analysis (PCA) by Singular Value Decomposition (SVD) of statistical datasets, we were able to identify the relevant properties of the hologram that determine the FWHM of the targeted area.

Scattering and optical aberrations through brain tissue are the dominant factor affecting spatial resolution. With a Gaussian model for scattering, we estimated the following relationship between the radial (Supplementary Figure 8a) and axial (Supplementary Figure 8b) FWHM and scattering depth, T.

$$\text{FWHM (Radial)} = 9.0\,\mu\text{m} * exp\left[\frac{T\,(\mu\text{m})}{9.8 * 10^2\,\mu\text{m}}\right] \tag{42}$$

$$\text{FWHM (Axial)} = 16.3\,\mu\text{m} * exp\left[\frac{T\,(\mu\text{m})}{1.8 * 10^3\,\mu\text{m}}\right] \tag{43}$$

The second most significant coefficient affecting spatial resolution is depth. This phenomenon is common in 3D holography setups and corresponds to asymmetric optical properties of the effective magnification numerical aperture of microscope objectives above and below the native focal plane.Using SVD, we estimate that :

$$\text{FWHM (Radial)} = 12.2\mu\text{m} + 8.7 * 10^{-3}\,x(\mu\text{m}) + 3.9 * 10^{-3}\,y(\mu\text{m}) + 1.1 * 10^{-2}\,z(\mu\text{m}) \tag{44}$$

$$\text{FWHM (Axial)} = 20.1\mu\text{m} + 5.5 * 10^{-4}\,x(\mu\text{m}) + 2.7 * 10^{-3}\,y(\mu\text{m}) + 2.2 * 10^{-2}\,z(\mu\text{m}) \tag{45}$$

In first approximation, this model reduces to :

$$\frac{\text{d}}{\text{d}z}\,(\text{FWHM (Radial)}) = 1.1 * 10^{-2} \qquad \frac{\text{d}}{\text{d}z}\,(\text{FWHM (Axial)}) = 2.2 * 10^{-2} \tag{46}$$

We note that characterization of how target location within the hologram affects the spatial resolution in a dataset where various amounts of optical scattering have been considered does not allow us to evaluate the hologram properties independently from optical aberrations, we therefore conducted a separate set of recordings without optical scattering through brain tissue to better evaluate this property.

Supplementary Note 8. Spatial resolution with large and dense 3D distributions of targets.

3D SHOT performance degradation through thick brain tissue compares to similar photostimulation instruments that rely on two-photon absorption with temporal focusing for enhanced depth selectivity, and the operational range is defined for targets no deeper than 500 µm in mouse brain tissue. Therefore, the next goal is to determine what other factors affect spatial resolution and how many neurons within this depth range can be reliably photo-stimulated.

The following results have been obtained through a very sensitive thin fluorescent film of fluorescent paint (TS-36 fluorescent Tamiya Color for plastics) sprayed on a clear microscope slide,

allowing us to characterize the performance limitations of 3D-SHOT independently of laser power considerations. In practice, we emphasize that laser power currently remains the limiting factor for simultaneous single shot photo activation of multiple neurons with present opsin technology.

We considered several holograms with variable numbers of targets (between 20 and 750), and with variable target density by scaling the point cloud around the center of the target area.

Statistical results are shown in Supplementary Figure 9 where we display the radial and axial FWHM as a function of target location (x,y,z) (Supplementary Figure 9a,c) and by color coding tour data with respect to the distance to the nearest target in the hologram, which characterizes the local density of targets.

We find (Supplementary Figure 9b,d) that the local density of targets is not a relevant parameter affecting FWHM. However, as observed through scattering tissue, we observe that FWHM depends on the target location.

We used SVD to parametrize the dependence from statistical data and estimate that :

$$\text{FWHM (Radial)} = 8.6\text{µm} - 1.7 * 10^{-3}\, x(\text{µm}) + 8.0 * 10^{-4}\, y(\text{µm}) - 1.0 * 10^{-3}\, z(\text{µm}) \tag{47}$$

$$\text{FWHM (Axial)} = 14.4\text{µm} - 2.1 * 10^{-3}\, x(\text{µm}) + 4.2 * 10^{-3}\, y(\text{µm}) + 1.8 * 10^{-2}\, z(\text{µm}) \tag{48}$$

We find similarly, that spatial dependence of the FWHM, observed independently from the presence of optical scattering simplifies to :

$$\frac{\mathrm{d}}{\mathrm{d}z}\left(\text{FWHM (Axial)}\right) = 1.8 * 10^{-2} \tag{49}$$

which is most likely related to the non-symmetrical numerical aperture and magnification properties on either side of the objective's focal plane. Overall, these results confirm that spatial resolution does not significantly degrade based on the physical location of targets within a given hologram, as long as they remain within the operating volume determined by both the optical system's magnification, and by the SLM's pixel density.

Finally, the ability of 3D SHOT to precisely trigger action-potentials relates to precise timing of illumination and to the characteristic response of the photosensitive opsin. The speed at which different sets of neurons can be activated in a sequence is defined by the the refresh rate of the SLM (60Hz with our hardware, however faster SLMs are now commercially available.) For each hologram displayed on the SLM, 3D-SHOT can simultaneously illuminate multiple targets with a single hologram on the SLM, and the photo-excitation timing of simultaneously targeted neurons is then only limited by our ability to gate the photo-excitation laser (Supplementary Figure 10).

Supplementary Note 9. Physiological spatial resolution with multi-target holograms.

To evaluate the ability of 3D-SHOT to photoactivate a specific set of neurons without inducing action-potentials anywhere else within the volume of interest, we simulate a piece of mouse brain tissue by considering a set of randomly distributed virtual neurons with a spatial density that compares to typical L2/3 barrel cortex. We then simulate a 3D-SHOT hologram targeting a specific subset of neurons within this volume, and for each neuron in the volume of interest, we compute the probability to spike given the 3D physiological point spread functions of the 3D-SHOT targets, which we treat as independent.

The simulation is repeated 100 times with randomly seeded 3D-SHOT targets on each trial. Simulation results are shown in Supplementary Figure 11a. 1,50, and 300 targeted neurons (in red) are intermixed within a dense network of non targeted neurons. Extreme spatial confinement of the two-photon absorption, combined with the natural threshold response of neurons indicates that 3D-SHOT could enable single neuron spatial resolution for up to 300 targets when laser power and opsin sensitivity can be made compatible for activating this number of simultaneous targets. While increasing the number of 3D-SHOT targets increases the number of action potentials generated from off-target neurons, most of these action potentials are expected to arise from neurons with a low probability of firing; that is, relatively few off-target neurons will be reliably driven by any given 3D-SHOT stimulus.
